# Cancerin: A computational pipeline to infer cancer-associated ceRNA interaction networks

**DOI:** 10.1371/journal.pcbi.1006318

**Published:** 2018-07-16

**Authors:** Duc Do, Serdar Bozdag

**Affiliations:** Department of Mathematics, Statistics, and Computer Science, Marquette University, Milwaukee, Wisconsin, United States of America; Ottawa University, CANADA

## Abstract

MicroRNAs (miRNAs) inhibit expression of target genes by binding to their RNA transcripts. It has been recently shown that RNA transcripts targeted by the same miRNA could “compete” for the miRNA molecules and thereby indirectly regulate each other. Experimental evidence has suggested that the aberration of such miRNA-mediated interaction between RNAs—called competing endogenous RNA (ceRNA) interaction—can play important roles in tumorigenesis. Given the difficulty of deciphering context-specific miRNA binding, and the existence of various gene regulatory factors such as DNA methylation and copy number alteration, inferring context-specific ceRNA interactions accurately is a computationally challenging task. Here we propose a computational method called Cancerin to identify cancer-associated ceRNA interactions. Cancerin incorporates DNA methylation, copy number alteration, gene and miRNA expression datasets to construct cancer-specific ceRNA networks. We applied Cancerin to three cancer datasets from the Cancer Genome Atlas (TCGA) project. Our results indicated that ceRNAs were enriched with cancer-related genes, and ceRNA modules in the inferred ceRNA networks were involved in cancer-associated biological processes. Using LINCS-L1000 shRNA-mediated gene knockdown experiment in breast cancer cell line to assess accuracy, Cancerin was able to predict expression outcome of ceRNA genes with high accuracy.

## Author summary

CeRNA interaction is a post-transcriptional gene regulation that involves interactions between RNAs competing for common miRNA regulators. Dysregulation of ceRNA interactions have been implicated in multiple diseases including cancer. Here we propose a computational pipeline called Cancerin that infers genome-wide ceRNA interactions in cancer. Unlike existing ceRNA inference tools that consider miRNAs as the only factor that regulate gene expression, Cancerin considers other types of gene regulators besides miRNAs, namely transcription factors, copy number alteration, and DNA methylation. To identify miRNA regulators for each gene, Cancerin incorporates a LASSO-based variable selection procedure that leverages both sequence-based and gene expression information. Then multiple expression-based filtering conditions are employed to select ceRNA interactions. Cancerin was applied to three cancer datasets from TCGA. Functional analysis indicated that the inferred ceRNAs were enriched with cancer-related genes, and ceRNAs within ceRNA modules (densely-connected ceRNAs) were involved in cancer-associated biological processes. Survival analysis showed that compared to non-ceRNAs, ceRNAs hold better prognostic power to predict survival outcomes. Our results showed that Cancerin can be used to identify genome-wide and functionally important ceRNA interactions, which makes it a valuable tool to better understand this recently discovered gene regulation mechanism and its role in cancer biology.

This is a *PLoS Computational Biology* Methods paper.

## Introduction

MicroRNAs (miRNAs) are a family of short non-coding RNA molecules involved in post-transcriptional gene regulation. MiRNAs attach to Argonaute protein to form RNA-induced silencing complexes (RISCs), which bind to miRNA-response-elements (MREs) located on the 3′UTR of messenger RNAs (mRNAs). This binding promotes mRNA degradation or inhibit their translation into proteins [1]. A typical mRNA contains multiple MREs, which are binding sites for one or multiple miRNAs. Thus, a mRNA can be targeted by multiple miRNAs, and a miRNA can target multiple mRNAs [2].

As protein synthesis is impacted by miRNA-mRNA binding, gene regulation by miRNAs plays an important role in a wide range of biological processes such as cell growth, differentiation, and apoptosis [3–5]. Anomaly in miRNA regulation have been implicated in multiple diseases including cancer [6]. Aberrant changes in miRNA concentration in cells could lead to dysregulation of tumor suppressors or oncogenic genes, which could trigger cancer development and progression [7].

Recent experimental studies suggest a new layer of miRNA-mediated regulation that involves indirect interactions between RNA molecules via their interactions with common miRNAs [8, 9]. Such RNAs are called competing endogenous RNAs (ceRNAs), and their indirect interactions are referred as ceRNA interactions [8]. The ceRNA hypothesis [10] posits that change of expression level in one ceRNA would alter its miRNA regulators’ abundance, which in turn alters the expression level of other target ceRNAs of these miRNAs. For example, a highly expressed ceRNA can sequester many miRNA molecules, reducing the total miRNA abundance and leading to the derepression of other target ceRNAs of these miRNAs. CeRNA interactions are not only among protein coding RNAs (i.e., mRNAs). Recent studies have found that non-coding RNAs (e.g., long non-coding RNAs (lncRNAs) [11, 12] and pseudogenes [13]) also involve in ceRNA interactions. For the rest of the paper, “RNAs” refers to candidate ceRNAs, which includes mRNAs and lncRNAs. CeRNA interactions have been shown to regulate important biological processes such as muscle differentiation [9], self-renewal capability of embryonic stem cells [3], and inhibition of cancer cell differentiation [14]. Disruption of ceRNA interactions has been implicated in multiple types of diseases including cancer [15, 16]. Disruption of ceRNA interactions can repress tumor-suppressor genes and lead to oncogenic activities [17, 18]. Comprehensive reviews of functions of ceRNA in cancer biology can be found in [19–21].

The existence and strength of ceRNA interactions may vary significantly in different physiological and cellular settings (i.e., normal cells versus tumor cells). As ceRNA interaction is considered as a new layer of gene regulation, identification and construction of genome-wide and condition-specific ceRNA interaction networks could facilitate better understanding of ceRNA regulatory mechanisms and their biological significance. While experimental studies are of great importance to confirm ceRNA interactions, inference of ceRNA interaction networks by only experimental methods would be time- and cost-prohibitive. Thus, computational tools are needed to infer ceRNA interaction networks and generate new hypotheses for further experimental validation.

Since ceRNA interactions are mediated via miRNAs, identifying interactions between miRNAs and their targets is a prerequisite to infer ceRNA interactions. Sequence-based miRNA target prediction algorithms such as TargetScan [22] and miRanda [23] have been employed to search for MREs in 3′UTR of mRNAs, and miRNA-mRNA interaction databases such as starBase [24] and miRWalk [25] store computationally and experimentally verified miRNA-mRNA interactions. Expression profiles of both mRNAs and miRNAs were also used to identify condition-specific miRNA-mRNA interactions. As miRNAs were mostly known to repress the expression of its targets, expression levels of miRNAs and their targets were often required to be negatively correlated [26, 27].

After predicting miRNA-target gene interactions, existing ceRNA inference methods differed in how they related expression of miRNAs and their co-regulated genes to decide which genes can establish ceRNA interactions. Pairwise gene expression correlation was often considered as the main criterion to select ceRNA interactions. Two ceRNAs were required to have positively correlated expression, and the ceRNAs and their miRNA regulators were required to have negatively correlated expression [26, 27]. However, miRNA expression data were also used to directly model the mediating effect of miRNAs in regulating ceRNA interaction. Partial Pearson correlation (PPC) [28] and conditional mutual information (CMI) [29, 30] metrics have been used to measure linear or nonlinear dependence of candidate ceRNAs’ expression on their shared miRNAs’ expression. Applying CMI to identify and construct a glioblastoma-specific ceRNA interaction network, Sumazin et al. found experimentally validated interactions between PTEN and their known ceRNAs in the ceRNA network [29]. In [28], a new metric called sensitivity partial correlation was proposed to quantify the expression correlation dependency between two ceRNAs conditioned on their shared miRNAs’ expression. The researchers applied this metric to gene and miRNA expression of normal and tumor breast samples to construct normal-specific and tumor-specific ceRNA interaction networks. They observed that multiple cancer hallmarks such as tumor inflammation were only enriched in the tumor-specific ceRNA network. A detailed review on computational methods to infer ceRNA interactions can be found in [31].

In existing ceRNA studies, most computational methods consider miRNAs as the only type of gene regulators, while overlooking other important types of gene regulators such as transcription factors, DNA methylation, and copy number alteration. Not considering other types of regulators might lead to spurious miRNA-gene interactions, which would cause false positive predictions of ceRNA interactions. Notably, lack of experimental studies to confirm ceRNA interactions posed a big challenge to validate the accuracy and significance of inferred ceRNA interactions.

This study presents a computational pipeline called Cancerin, which infers ***Can***cer-associated ***ceR***NA ***i***teraction ***n***etworks. A cancer-associated ceRNA interaction is defined as an interaction between two differentially expressed RNAs (between normal and cancer samples), and the interaction is mediated by some differentially expressed miRNAs that regulate both RNAs. Besides mRNAs, non-coding RNAs such as long non-coding RNAs (lncRNAs) have been shown to actively participate in functionally important ceRNA interactions in both normal and cancer cells [19, 21]. Thus, our pipeline considers both mRNAs and lncRNAs as potential ceRNAs. To infer interaction between miRNAs and their RNA targets (i.e., candidate ceRNAs), Cancerin employs knowledge from both putative miRNA-RNA interactions and miRNA/RNA expression profiles. In addition, Cancerin incorporates other types of gene expression regulatory factors, namely copy number alteration, DNA methylation, and transcription factors to infer miRNA-RNA interactions, which distinguish Cancerin from existing ceRNA inference methods. An easy-to-use R software for Cancerin is freely available at https://github.com/bozdaglab/Cancerin.

Cancerin was applied to three cancer datasets. Our result indicated that the ceRNAs in the obtained ceRNA interaction networks were significantly enriched with cancer-related genes. Additionally, we observed that closely connected ceRNAs in the ceRNA networks were associated with cancer cell formation and development processes. Compared to non-ceRNA genes, we showed that expression change of predicted ceRNAs had higher association with cancer survival outcomes. To validate the effect of ceRNA interactions to expression change on an external dataset, we used the LINCS perturbation dataset [32] and observed that knockdown of ceRNAs was associated with the expression change of their ceRNA partners.

## Materials and methods

### Datasets

We used the R Bioconductor package TCGABiolinks [33] to download genomic and clinical data of normal and solid tumor tissues for three types of cancer from The Cancer Genome Atlas (TCGA) [34]. Cancer types were breast invasive carcinoma (BRCA), kidney renal clear cell carcinoma (KIRC), and head and neck squamous cell carcinoma (HNSC). We retrieved level 3 data for raw count mRNA and miRNA expression (Illumina HiSeq 2000), copy number alteration (Affymetrix SNP Array 6.0), and DNA methylation level (Infinium HumanMethylation450 Bead-Chip). The expression of lncRNAs was retrieved from the TANRIC database [35]. We only kept tissue samples for which all of these genomic data and clinical data were available. In addition, 3′UTR sequences of 18,959 mRNAs and 13,870 lncRNAs were downloaded from the GENCODE Release 26 (GRCh38.p10) [36], and sequences of 2,588 mature miRNAs were downloaded from miRBase release 21 [37]. Putative miRNA-mRNA interactions were retrieved from starBase v2.0 [24] and TargetScan 7.1 [22] databases. Putative miRNA-lncRNA interactions were retrieved from starBase v2.0 [24], DIANA-LncBase v2 [38], and LnCeDb [39]. Putative TF-gene interactions were retrieved from the TRED [40] and TRRUST (version 2) [41] databases.

### Data preprocessing

#### Gene expression processing and differential expression analysis

To filter out low-count RNAs, we used the R Bioconductor package edgeR [42] to convert raw counts of mRNAs and miRNAs to CPM (counts-per-million) values. RNAs that were not expressed in the majority of samples were filtered out. Specifically, across all the samples for each cancer dataset, an RNA was filtered out if its CPM value was less than 1 in more than *t* samples, where *t* was set to the larger between the tumor and the normal group size.

To identify differentially expressed (DE) mRNAs and DE miRNAs between normal and tumor samples, we employed the R package edgeR [42]. EdgeR normalizes the raw data using TMM (trimmed means of M values) method and models count data with negative binomial (NB) distribution. After normalizing the data and fitting it under NB models, we applied exact test [42] to identify DE mRNAs and DE miRNAs. As expression of lncRNAs was in RPKM units and was normalized to follow a normal distribution, to find DE lncRNAs, we fitted a linear model for each lncRNA using the lmFit function in the R package limma [43]. A miRNA, mRNA, or lncRNA was considered to be differentially expressed if its adjusted Bonferroni-Hochberg p-value [44] was smaller than 0.01.

To ensure the expression of the DE mRNAs, miRNAs, and lncRNAs is in the same units, we converted raw counts of DE mRNAs and DE miRNAs to RPKM. We used log2(RPKM+0.001) to present the expression of all DE RNAs. The expression of those RNAs were z-normalized across all the tumor samples as we only used the tumor samples in the subsequent steps.

#### Copy number alteration

Level 3 copy number alteration data from TCGA provided estimated mean copy numbers of chromosomal segments in the whole genome. Using the genomic location information of 22,310 protein coding genes provided by GENCODE Release 26 (GRCh38.p10), we applied the R Bioconductor package CNTools [45] to convert the segmented CNA data into a gene-level data matrix where each entry represented copy number value of a gene in a specific sample.

#### DNA methylation

Level 3 DNA methylation data from TCGA samples measured the methylation level of approximately 450,000 CpG sites genome-wide. The methylation level of each CpG site (i.e., *β* value) was estimated as the ratio of the methylated probe intensity to the overall intensity (sum of methylated and unmethylated probe intensities). Thus *β* ranges between 0 and 1, with 0 being hypomethylated and 1 being hypermethylated. Previous studies [46, 47] indicated that the methylation of CpG sites in promoter regions were associated with gene expression change. Therefore, we only considered *β* values of CpG sites in genes’ promoter regions. Thus, to compute gene-centric methylation values, we used the Bioconductor annotation package IlluminaHumanMethylation450kanno.ilmn12.hg19 [48] to identify the probes positioned at the upstream 200 to 1500 base pairs from of gene transcription start site. A gene’s methylation level was estimated as the mean of its associated upstream probes’ *β* values.

### Cancerin pipeline

**Fig 1:**
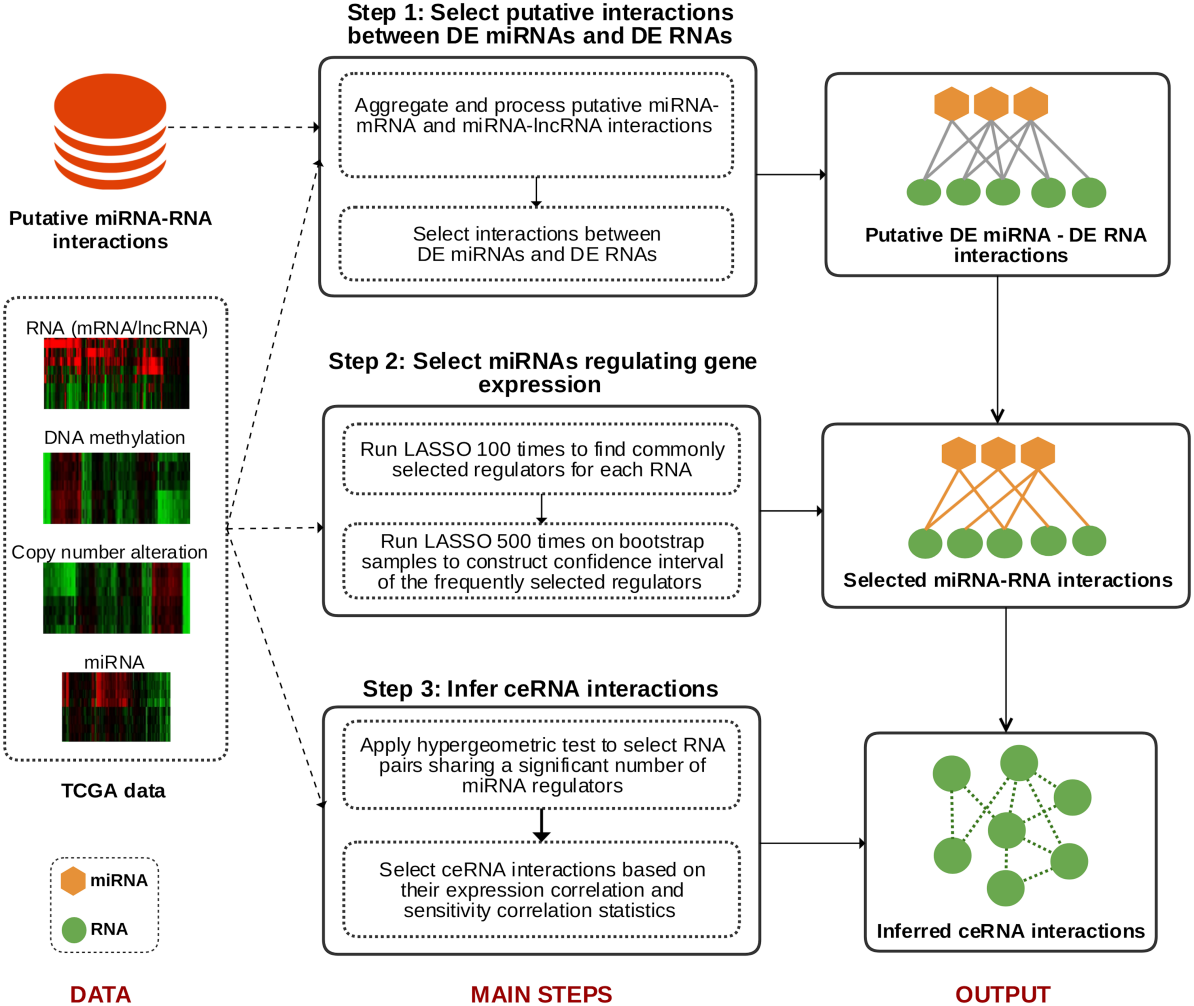
Cancerin pipeline to infer cancer-associated ceRNA interaction networks. Cancerin consists of three main steps. In step 1, for each DE RNA, Cancerin selects its candidate DE miRNA regulators based on sequence binding results. In step 2, Cancerin applies a LASSO-based variable selection procedure to select a subset of miRNA regulators that contribute to the expression variation of the DE RNA. In step 3, Cancerin applies multiple filtering conditions to infer ceRNA interactions between the RNAs that are regulated by common miRNAs.

Cancerin is a computational pipeline to identify genome-wide cancer-associated ceRNA interaction networks. It consists of three main steps. Using putative miRNA-mRNA and miRNA-lncRNA interactions, the first step aims to construct an interaction network between DE miRNAs and DE RNAs. In the second step, only the miRNAs that are associated with their targeted RNAs’ expression change are kept. In the final step, several filtering layers are applied to infer ceRNA interactions between RNAs that are targeted by common miRNAs. The entire Cancerin pipeline is illustrated in Fig 1. The details in each step in Cancerin are described in the following.

#### Identifying putative regulatory interactions between DE miRNAs and DE mRNAs based on sequence binding

Putative interactions between DE miRNAs and DE mRNAs in humans were retrieved from the TargetScan 7.1 [22] and starBase v2.0 [24] databases. TargetScan assigns an mRNA to be a miRNA’s target if the mRNA contains conserved 8mer, 7mer, and 6mer sites that are complementary to the seed regions of the miRNA. starBase stores miRNA-RNA interactions predicted by analyzing 108 CLIP-seq datasets. After aggregating all putative interactions from the two databases, we applied the miRanda algorithm [23] to select only the miRNA-mRNA pairs such that there existed at least one MRE on the 3′UTR of the mRNA that was complementary to the miRNA sequence.

We retrieved putative interactions between DE miRNAs and DE lncRNAs from starBase v2.0 [24], DIANA-LncBase v2 [38] and LnCeDb [39]. The predicted miRNA-lncRNA interactions in DIANA-LncBase v2 are inferred using DIANA-microT algorithm [49], which is a machine-learning approach that estimates miRNA-RNA target binding score base on weighting multiple features such as sequence complementarity, free binding energy and conservation profile. The putative miRNA-lncRNA interactions in LnCeDb come from two sources: interactions from Mircode database [50], which used seed complementarity and evolutionary source to infer interactions, and interactions inferred by its own sequence-based miRNA-RNA target prediction algorithm.

#### Selecting miRNAs associated with expression change of their predicted RNA targets

For each DE RNA and its putative DE miRNA regulators (selected in the previous step), Cancerin identified which miRNAs contributed to the RNA’s expression variation. It is well known that beside miRNA regulation, RNA expression can be controlled by other factors such as its transcription factors (TFs), copy number alterations (CNA), and DNA methylation (DM) [51]. A procedure to identify regulatory interactions between miRNAs and its RNA targets should also take other types of gene regulators into account. Thus, our LASSO-based variable selection procedure to infer cancer-specific miRNA-mRNA interactions incorporated additional types of gene regulators including TF, CNA, and DM.

LASSO is a regularized regression method that penalizes the sum of absolute value of the regression coefficients, so that it shrinks some covariates’ coefficients to be exactly zero. Hence, it can be used for variable selection purposes [52]. LASSO regression was applied for each RNA. For each mRNA, its expression was used as the response variable’s value and its CNA, DNA methylation, and the expression of its candidate miRNAs and TFs were used as independent variables’ values. For each lncRNA, its expression was used as the response variable’s value and its candidate miRNAs’ expression were used as the independent variables’ value. As mentioned in the data preprocessing section, we only used tumor samples in this (and subsequent) analysis.

Training a LASSO model requires selecting the regularization hyperparameter λ. To select the optimal λ value, we applied 10-fold cross validation to find the λ value that provided the simplest model such that its cross-validation error was within one standard error of the minimum cross-validation error. Thus, for each *RNA*_j_, out of all of its candidate predictors (independent variables), LASSO regression selected a set of non-zero coefficient predictors. We employed R package HDCI [53] to perform LASSO regression.

However, independent variables selected by LASSO have been shown to be inconsistent especially when sample size gets large [54]. To address this problem, we ran the LASSO regression 100 times for each RNA. Only the non-zero coefficient predictors that were selected more than 75 times were considered as frequently selected regulators of the RNA.

Unlike in linear multiple regression where each independent variable’s regression coefficient is associated with a p-value testing the null hypothesis that its coefficient is equal to zero, coefficients of LASSO-selected predictors are not associated with any statistical significance test. To address this problem, we employed a bootstrap procedure to construct a confidence interval for the frequently selected predictors that were obtained above. Suppose a regulator *R*_i_ is a frequently selected predictor for *RNA*_j_. From the 100 LASSO runs, we used the median of *R*_i_’s coefficients to represent its regression coefficient and called it ${\bar{\alpha }}_{\text{ij}}$. To estimate the confidence interval of ${\bar{\alpha }}_{\text{ij}}$, for the *RNA*_j_, we fitted LASSO regression 500 times, each time to a set of bootstrapped samples, to generate a bootstrap regression coefficient distribution {*α*_bootstrap_ij_}. *R*_i_ would be kept as one of the *RNA*_j_’s regulators if its ${\bar{\alpha }}_{\text{ij}}$ was within the 95% confidence interval of {*α*_bootstrap_ij_} and the 95% confidence interval did not include 0. As miRNAs are mostly known to repress the expression level of its RNA target, for each RNA, out of all the kept variables, we only selected the miRNAs that had negative ${\bar{\alpha }}_{\text{ij}}$ coefficients.

#### Identifying cancer-associated ceRNA interaction network

Using the miRNA-RNA interactions obtained in the previous step, we generated all possible RNA-RNA pairs such that the constituent RNAs in each pair share at least one miRNA regulator. Those pairs were considered as candidate ceRNA pairs. Following the ceRNA hypothesis, we only kept the candidate ceRNA pairs with high positive Pearson expression correlation (correlation ≥ 0.5, p-value < 0.05).

Given the number of miRNAs regulating each RNA, to assess whether the two RNAs in each candidate ceRNA pair shared a significant number of miRNA regulators, we applied a hypergeometric test on each of the candidate ceRNA pairs. Let *N* be the total number of all DE miRNAs. For a ceRNA pair consisting of *RNA*_*i*_ and *RNA*_*j*_, let *N*_*i*_ and *N*_*j*_ be the total number of miRNAs regulating *RNA*_*i*_ and *RNA*_*j*_, respectively, and *N*_*ij*_ be the number of common miRNAs regulating both *RNA*_*i*_ and *RNA*_*j*_. The p-value of the hypergeometric test was calculated using the formula in Eq 1. Based on the hypergeometric test results, a candidate ceRNA pair was selected if its adjusted Bonferroni-Hochberg p-value was smaller than 0.05.

$$\begin{array}{c} p-value=1-\sum_{k=0}^{N_{ij}-1}\frac{\left(\begin{array}{c} N_{j}\\ k \end{array}\right)\left(\begin{array}{c} N-N_{j}\\ N_{i}-k \end{array}\right)}{\left(\begin{array}{c} N\\ N_{i} \end{array}\right)} \end{array}$$ (1)

To further eliminate potentially spurious ceRNA pairs, we employed the sensitivity correlation (SC) metric proposed in [28] to estimate the ceRNA interaction strength for each ceRNA pair. Let {*miRNA*_*ij*_} be the set of common miRNAs regulating both *RNA*_*i*_ and *RNA*_*j*_. Let *Corr*(*RNA*_*i*_, *RNA*_*j*_) be the expression correlation between *RNA*_*i*_ and *RNA*_*j*_ and *PC*(*RNA*_*i*_, *RNA*_*j*_|{*miRNA*_*ij*_}) be the partial expression correlation between *RNA*_*i*_ and *RNA*_*j*_ conditioned on {*miRNA*_*ij*_}. Sensitivity correlation *SC*(*RNA*_*i*_, *RNA*_*j*_|{*miRNA*_*ij*_}) is defined in Eq 2:

$$\begin{array}{c} \begin{array}{c} SC\left(RNA_{i},RNA_{j}|miRNA_{ij}\right)=Corr\left(RNA_{i},RNA_{j}\right)\\ -PC(RNA_{i},RNA_{j}|\left\{miRNA_{ij}\right\}) \end{array} \end{array}$$ (2)

The R package bnlearn [55] was used to compute partial correlation (PC) for each candidate ceRNA pair. Since *PC*(*RNA*_*i*_, *RNA*_*j*_|{*miRNA*_*ij*_}) computed the correlation of the RNAs’ expression while controlling/eliminating the effect of their shared miRNAs’ expression, *SC*(*RNA*_*i*_, *RNA*_*j*_∣{*miRNA*_*ij*_}) quantifies the contribution of the shared miRNAs to the linear relation between the expression of the two RNAs. A high SC value signifies a strong indirect interaction between the two RNAs mediated by shared miRNA regulators. Thus, we selected the ceRNA pairs with positive SC values and their p-values from partial correlation test smaller than 0.05. Additionally, to estimate the statistical significance of SC, we computed the SC empirical p-value for each candidate ceRNA pair. For the pair (*RNA*_*i*_,*RNA*_*j*_), suppose the {*miRNA*_*ij*_} was of size *N*_*ij*_, then we randomly selected *N*_*ij*_ miRNAs to compute the pair’s sampled *SC* value. For each ceRNA pair, the resampling procedure was repeated 1000 times. An empirical SC p-value was assigned as the percentage of iterations in which the sampled SC value exceeded the original SC value. A ceRNA pair was kept if its empirical SC p-value was smaller than 0.05.

## Results

Cancerin pipeline leveraged multidimensional cancer genomics data to infer cancer-associated ceRNA interaction networks. In order to assess Cancerin, we used Cancerin to infer ceRNA networks in three cancer types, namely breast (BRCA), kidney (KIRC), and head and neck cancer (HNSC). We obtained the RNAseq, miRNAseq, DNA methylation, and CNA datasets for BRCA, KIRC and HNSC samples from TCGA [34]. The numbers of normal/tumor tissue samples in each cancer type were 47/193 (BRCA), 20/243 (KIRC), and 20/413 (HNSC).

### Putative interactions between DE miRNAs and DE RNAs

The first step in Cancerin involved aggregating the putative interactions between miRNAs and RNAs from various data sources. The candidate miRNA-mRNA interactions were downloaded from the starBase and the TargetScan databases. Using mRNAs’ and miRNAs’ FASTA sequences, we selected only the mRNAs whose 3′UTR sequences and the miRNAs whose mature sequences were specified. To further refine those putative interactions, the miRanda algorithm was used to check for the existence of MRE(s) on the mRNAs’ 3′UTR and to estimate the thermodynamic folding energy between the miRNAs and their predicted mRNA targets. The lower the energy, the higher chance that an interaction will actually occur [56]. A miRNA-mRNA interaction was kept if there existed at least one MRE on the mRNA as miRNA’s binding site and the miRNA-mRNA interaction’s folding energy was lower than 140 kcal/mol (default value). After applying miRanda, there remained 465,049 interactions between 473 miRNAs and 13,932 mRNAs. Putative miRNA-lncRNA interactions were aggregated from starBase v2.0, DIANA-LncBase v2, and and LnCeDb, resulting in 3,961,135 interactions between 2,695 miRNAs and 24,215 lncRNAs.

Given all the putative miRNA-RNA interactions, we only kept the interactions between DE miRNAs and DE RNAs. Table 1 summarizes the number of DE miRNA—DE RNA interactions in each cancer type.

**Table 1:** Number of putative DE miRNA-DE RNA interactions and number of DE miRNAs and DE RNAs included in those interactions (output for Cancerin—Step 1).

	BRCA	KIRC	HNSC
No. of putative DE miRNA—DE mRNA interactions	153,465	107,348	94,980
No. of DE miRNAs ^1^	215	164	201
No. of DE mRNAs ^1^	7,502	6,690	5,005
No. of putative DE miRNA—DE lncRNA interactions	60,935	18,589	17,350
No. of DE miRNAs ^2^	215	164	201
No. of DE lncRNAs ^2^	3,111	1,335	896

^1^: included in putative DE miRNA—DE mRNA interactions.

^2^: included in putative DE miRNA—DE lncRNA interactions.

To identify cancer-associated ceRNA interactions, Cancerin employed the putative miRNA-RNA interactions and the RNA expression as input data for the next two steps, which included applying a LASSO-based variable selection procedure to select cancer-specific miRNA-RNA interactions and using that information to identify ceRNA interactions.

### Analysis of miRNA-RNA interactions obtained from the LASSO-based variable selection procedure

The LASSO-based variable selection procedure (see Materials and methods) was applied to identify cancer-specific miRNA-RNA interactions while also taking into account the other types of gene regulators including TF, DNA methylation, and CNA. Table 2 summarizes the number of miRNA-RNA interactions selected by the variable selection procedure in each cancer type. Details of the selected miRNA-RNA interactions could be found in S1 File.

**Table 2:** Number of selected miRNA-RNA interactions obtained after applying the variable selection procedure (output of Cancerin—Step 2).

	BRCA	KIRC	HNSC
No. of miRNA-mRNA interactions	6,616	8,408	9,893
No. of miRNAs ^1^	196	154	190
No. of mRNAs ^1^	2,814	2,971	3,020
No. of miRNA-lncRNA interactions	502	217	467
No. of miRNAs ^2^	134	93	141
No. of lncRNAs ^2^	210	91	175

^1^: included in the selected miRNA—mRNA interactions.

^2^: included in the selected miRNA—lncRNA interactions.

#### Many miRNA-RNA interactions were only identified when different types of gene expression regulators were taken into account

Cancerin pipeline was constructed under the premise that different types of gene regulators were important to correctly infer miRNA-RNA interactions. Out of all the RNA targets that were found to have at least one miRNA regulator (3,024 (BRCA), 3,062 (KIRC), and 3,195 (HNSC)), we computed the percentage of those targets that were also under regulation of at least one additional regulatory factor such as CNA, DNA methylation, or TF (Table 3). Not surprisingly, those additional regulatory factors, especially CNA, were observed to be associated with the expression change in majority of the target RNAs.

**Table 3:** Percentage of RNA targets regulated by miRNAs and also by at least one additional type of regulators.

	BRCA	KIRC	HNSC
Percentage of RNA targets under CNA regulation	76.2%	69.2%	77.2%
Percentage of RNA targets under DNA Methylation regulation	30.4%	26.3%	35.0%
Percentage of RNA targets under TF regulation	54.1%	59.3%	48.0%

To check the impact of those additional regulators in inferring miRNA-RNA interactions, we performed a comparative analysis between the miRNA-RNA interactions that were selected in two different cases depending on whether the different regulatory factors besides miRNA (i.e., CNA, DNA methylation, and TF) were present or not in the LASSO-based variable selection procedure. In the first case when those regulators were incorporated, we referred it as “Cancerin (original)”. The second case, in which miRNAs were the only type of regulators to be considered, was refereed as “Cancerin (only_miRNA)”. Table 4 shows the number of miRNA-RNA interactions and their constituent miRNAs and RNA targets selected in the two cases.

**Table 4:** Number of miRNA-RNA interactions and their constituent miRNAs and RNAs selected in “Cancerin (original)” and “Cancerin (only_miRNA)”. The first, second, and third value in each cell refers to the results from “Cancerin (original)”, “Cancerin (only_miRNA)”, and the common results between the two cases, respectively.

	BRCA	KIRC	HNSC
No. of miRNA-RNA interactions	7,118/4,071/3,242	8,625/6,524/5,085	10,360/8,648/6,619
No. of miRNAs	204/201/198	155/153/153	195/196/195
No. of RNAs	3,024/1,763/1,523	3,062/2,219/2,068	3,195/2,520/2,404

While the two cases selected similar miRNAs that have at least one RNA target (row 2 in Table 4), many miRNA-RNA interactions and RNA targets could only be found in “Cancerin (original)” (row 1 and 3 in Table 4). To check how the additional regulatory factors besides miRNAs played a role in that distinction, we looked at the common RNA targets that were included in both “Cancerin (original)” and “Cancerin (only_miRNA)”, and compared them with the RNA targets that were uniquely found in “Cancerin (original)”. Among the common RNA targets, the percentage of RNAs that had at least one additional regulator in “Cancerin (original)” results was 78.2% (BRCA), 83.8% (KIRC), and 85.2% (HNSC). Among the RNA targets unique to “Cancerin (original)”, the percentage values increased to 97.6% (BRCA), 96.7% (KIRC), and 97.1% (HNSC). These results suggest that while “Cancerin (only_miRNA)” could still discover some RNA targets that were regulated by an additional regulatory factor besides miRNAs, there were RNAs that could only be found to be regulated by miRNAs when different types of regulatory factors were incorporated in the variable selection step.

#### Hub miRNA regulators were known to be associated with cancer

In all three cancer types, there were miRNAs that regulated many RNA targets, which made those miRNAs common mediators in multiple ceRNA interactions. The miRNA regulators with highest number of RNA targets in each cancer type were let-7a-5p (BRCA), miR-106b-5p (KIRC), and miR-9-5p (HNSC), which contributed to 2.5%, 3.6%, and 2.5% of total miRNA-RNA interactions, respectively. Let-7a-5p was downregulated in the BRCA dataset (log fold change (FC) = -0.42, False Discovery Rate (FDR) = 7e-4). Known as a tumor-suppressor, let-7a-5p downregulation was shown to cause disruption of crucial signaling pathways including Janus protein tyrosine kinase (JAK) and signal transducer [57], which can lead to tumor cell migration and invasion in breast cancer [58, 59]. In the KIRC dataset, miR-106b-5p was upregulated (logFC = 1.5, FDR = 6e-19). Upregulation of this miRNA can enhance activation of PI3K signaling pathway and promote tumor cell metastasis in KIRC [60]. In the HNSC dataset, miR-9-5p was highly upregulated (logFC = 3.37, FDR = 5e-06). Upregulation of miR-9 family was known to activate oncogenic pathways in multiple cancers such as leukemia, breast, and colon cancer [61]. Interestingly, miR-130-3p was among the top five miRNAs that had highest number of RNA targets in all the three cancer types. Aberration in gene regulation by miR-130 family was known to drive tumorgenesis in many cancer types including BRCA, KIRC, and HNSC [62].

#### Selected miRNA-mRNA interactions included cancer-associated miRNA-mRNA interactions

To test if our variable selection procedure to identify miRNA-mRNA interactions was able to detect known cancer-associated miRNA-mRNA interactions, we retrieved 2,259 cancer-related miRNA-mRNA interactions from the oncomiRDB database [63]. Each miRNA-target interaction curated in oncomiRDB meets two conditions: (1) the miRNA is involved in at least one cancer-related phenotype or cellular process (2) the mRNA is a known oncogene or tumor-suppressor. As our method only used DE miRNAs and DE mRNAs as input, we only selected the interactions in oncomiRDB in which both miRNAs and mRNAs were also DE miRNAs and DE mRNAs.

We observed that several miRNA-mRNA interactions in the oncomiRDB database were also included in the miRNA-mRNA interactions inferred by Cancerin (step 2). We performed a hypergeometric test between the oncomiRDB interactions and inferred miRNA-mRNA interactions to test whether they shared a significant number of interactions. For each cancer type, the background sets in the hypergeometric test consisted of all possible pairs between DE mRNAs and DE miRNAs. The numbers of overlapping interactions and their p-values from the hypergeometric test in BRCA, KIRC, and HNSC were 50 (p-value = 1.75*E*^−39^), 40 (p-value = 4.6*E*^−24^), and 49 (p-value = 1.7*E*^−32^), respectively. We also performed the same hypergeometric test between the sequence-based miRNA-mRNA interactions (Cancerin—step 1) and the oncomiRDB interactions. The sequence-based interactions also had significant enrichment in oncomiRDB interactions (p-values ≈ 0 in all three cancer types).

### Analysis of inferred ceRNA networks

In Cancerin (step 3), given all the miRNA-RNA interactions obtained after applying the LASSO-based variable selection procedure, we identified all the candidate ceRNA interactions in which both the constituent RNAs were regulated by at least one common miRNA. Then we applied several filtering layers to select the final ceRNA interactions out of those candidate ceRNA pairs. Two RNAs were considered to have a ceRNA interaction if they had a significant number of shared miRNAs, and their expression profiles were both significantly correlated (correlation ≥ 0.5, p-value < 0.05) and had significantly positive sensitivity correlation (empirical p-value < 0.05). Table 5 summarizes the number of ceRNA interactions and the constituent ceRNAs in those interactions for each cancer type. Details of the selected ceRNA interactions could be found in S1 File.

**Table 5:** Number of inferred ceRNA interactions and number of ceRNAs in those interactions (output of Cancerin—Step 3).

	BRCA	KIRC	HNSC
No. of all ceRNA interactions	4,115	4,639	2,725
No. of mRNA-mRNA ceRNA interactions^1^	3,674	4,614	2,589
No. of mRNA-lncRNA ceRNA interactions^1^	394	25	121
No. of lncRNA-lncRNA ceRNA interactions^1^	47	0	15
No. of all ceRNAs	1,593	1,081	1,110
No. of mRNAs as ceRNAs^2^	1,491	1,071	1,063
No. of lncRNAs ceRNAs^2^	102	10	47

^1^: subset of all ceRNA interactions (Row 1)

^2^: subset of all ceRNAs (Row 5)

Overall, the selected ceRNA interactions were very specific to each cancer type. We found only one common ceRNA interaction in all the three cancer types. The number of common ceRNA interactions between any two cancer types was also very low (9 between BRCA and KIRC, 22 between BRCA and HNSC, and 32 between KIRC and HNSC). In all three cancer types, almost all ceRNA interactions were between mRNAs (84% (BRCA), 99% (KIRC), and 95% (HNSC)). In BRCA and HNSC, many lncRNAs that were involved in lncRNA-lnRNA ceRNA interactions also participated in mRNA-lncRNA ceRNA interactions. Specifically, out of 57 lncRNAs (BRCA) and 20 lncRNAs (HNSC) involved in lncRNA-lncRNA ceRNA interactions, 41 (BRCA) and 14 (HNSC) of those lncRNAs also participated in mRNA-lncRNA ceRNA interactions.

#### Inferred ceRNA networks were scale-free and independent from protein-protein interactions (PPI) and TF-gene interactions

**Fig 2:**
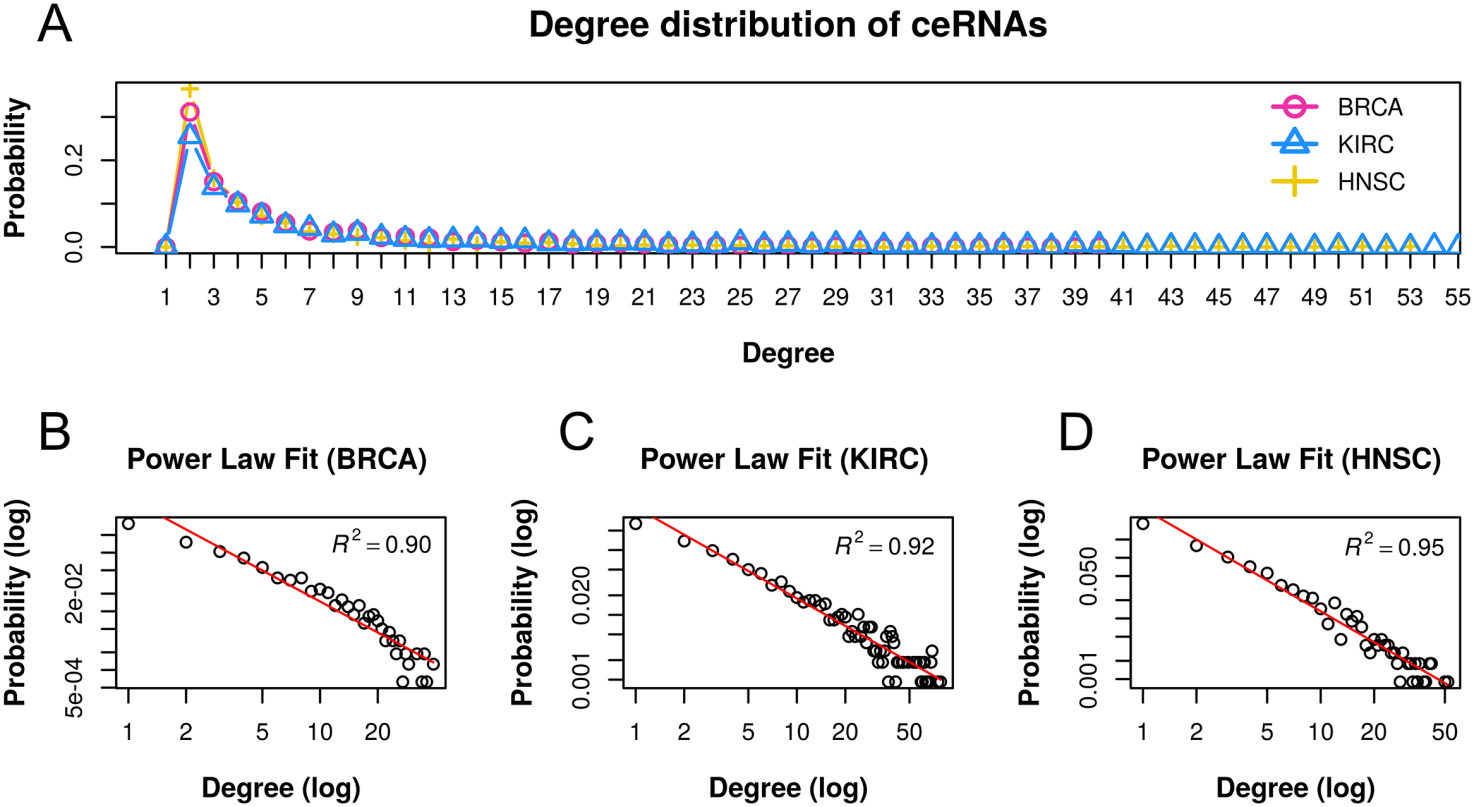
Degree distribution and power-law statistics. (A) Degree distribution of ceRNAs for each cancer type. Linear regression statistics between log(ceRNA’s degree) and log(ceRNA’s degree probability) in (B) BRCA, (C) KIRC, and (D) HNSC cancer types.

Biological networks usually exhibit scale-free property [64]. To check if the inferred ceRNA networks were scale-free, we computed the degree probability distribution function of each ceRNA network. Following the power-law rule [65], we fitted linear regression of log(ceRNA’s degree probability) to log(ceRNA’s degree). Log-log plots of all three ceRNA networks had negative slope with high fitness, which clearly indicated that the inferred ceRNA networks were scale-free (Fig 2).

Two genes can interact and thereby regulate each other via different regulatory layers (e.g., protein-protein interactions (PPIs) and TF-gene interactions). To test the specificity of Cancerin to identify ceRNA interactions, we checked whether the inferred ceRNA interaction networks also contained TF-gene interactions or PPIs. We collected 410,337 PPIs from BioGrid database version 3.4.159 [66]. Within the total number of inferred ceRNA interactions in each cancer network, very few interactions were PPI (0.85% (BRCA), 0.63% (KIRC), and 0.73% (HNSC)). Similarly, we also found very few ceRNA interactions that were also TF-gene interactions (0.78% (BRCA), 0.09% (KIRC), and 0.18% (HNSC)).

#### CeRNAs were significantly associated with cancer-related genes

To test if the ceRNAs in the inferred ceRNA networks were enriched in cancer-associated genes, we compiled a list of cancer-related genes (oncogenes and tumor-suppressor genes) from the Cancer Gene Census in COSMIC v83 [67], the Bushman lab’s Cancer Gene List v3 [68], and the Network of Cancer Genes 5.0 [69]. It resulted 2,944 cancer-related genes in total. We performed a hypergeometric test between the inferred ceRNAs in each cancer type with the cancer-related gene list. The results showed that ceRNAs were significantly enriched in the cancer-related genes (p-values were 4.3e-4 (BRCA), 5.0e-3 (KIRC), and 1.9e-5 (HNSC)). We also performed a hypergeometric test between the DE RNAs that were not predicted to be ceRNAs (i.e., non-ceRNAs) and the cancer-related genes. In all the three cancer types, unlike the ceRNAs, the non-ceRNAs did not show significant enrichment with the cancer-related genes (p-values ≈ 1 in all three cancer types).

To explore the significance of lncRNAs which were ceRNAs, we analyzed the degree of connection of lncRNAs in the ceRNA networks. A hub ceRNA in the network was defined as the ceRNAs which had high degree (i.e., top 90% degree) in the ceRNA network. Within of hub ceRNAs in each cancer, we found a small number of hub lncRNAs (11 (BRCA), 0 (KIRC), and 2 (HNSC)). Interestingly, MAGI2-AS3 was a hub lncRNA in both BRCA and HNSC, and it was also the lncRNA with the highest degree in both the BRCA and HNSC ceRNA interaction networks. Among the MAGI-AS3’s ceRNA partners, 25% (BRCA) and 35% (KIRC) of them were cancer-associated genes. Recently, MAGI2-AS3 was shown to play an important role in tumorigenesis and tumour progression in breast cancer [70]. These result suggests that while lncRNAs contributed to a small number of ceRNA interactions, the hub lncRNAs may hold important functions in cancer biology.

#### CeRNAs were potential biomarkers for cancer prognosis

In order to assess the prognostic power of the ceRNAs, we tested if the ceRNAs were better than the non-ceRNAs (i.e., DE genes not in the ceRNA network) at predicting survival status of cancer patients. Univariate Cox proportional hazard model was fit for each DE RNA, which was either a ceRNA or a non-ceRNA. The response variable was the number of days till death for each patient. The patients who were alive or had no death record were censored and their last follow-up dates were used.

After hazard model fitting, each DE RNA was associated with a hazard ratio and a p-value (from testing the null hypothesis that its hazard ratio equals to 1). A hazard ratio > 1 implies that an increase of expression of the gene increases the risk of death, while a hazard ratio < 1 implies that an increase of the gene expression decreases the risk of death. Thus, the prognostic power of a gene is reflected through how much its hazard ratio is deviated from 1 (i.e., ∣hazard ratio—1∣).

**Fig 3:**
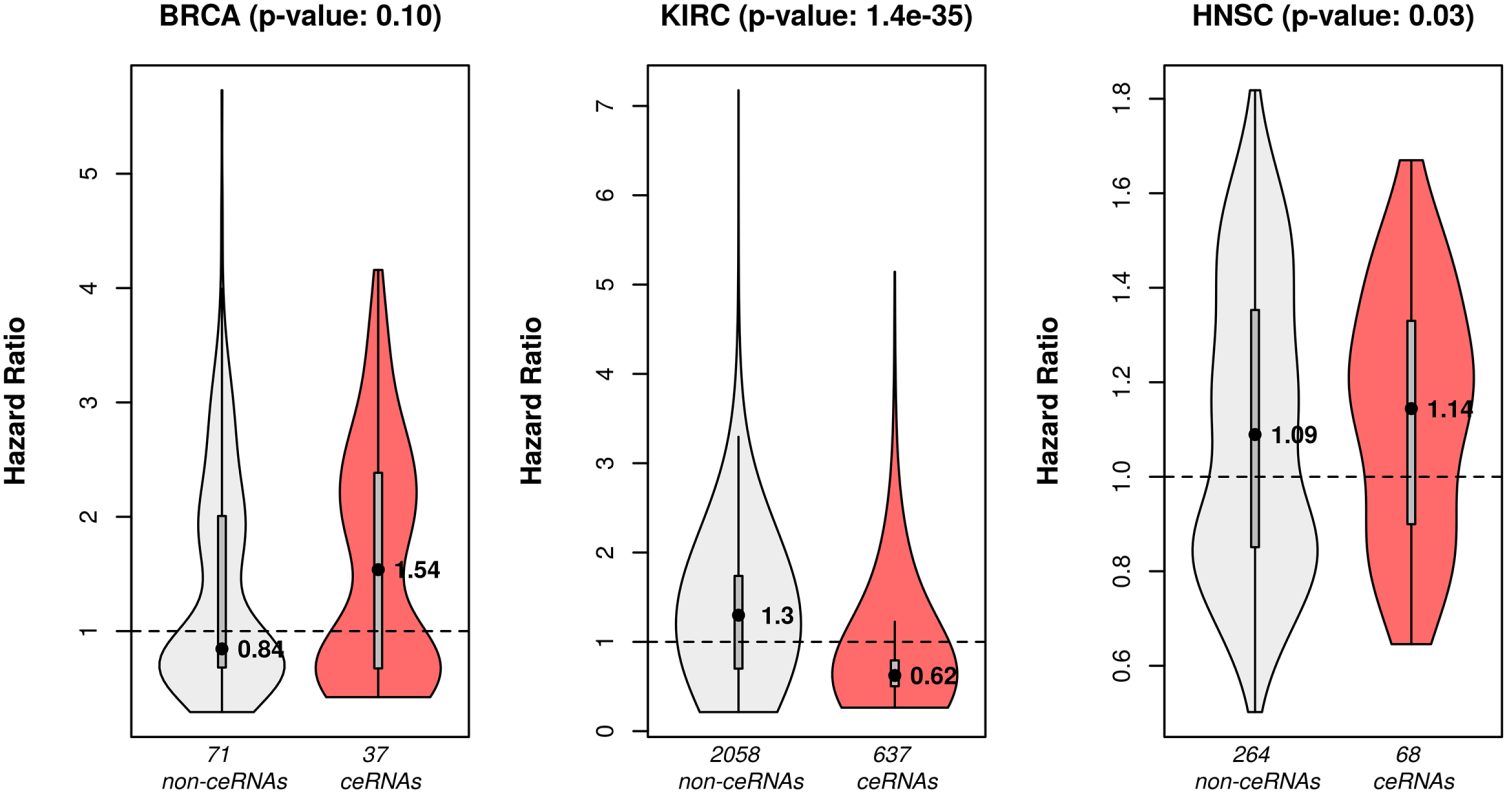
Hazard ratio distribution of prognostic ceRNAs and non-ceRNAs in each cancer type. A prognostic RNA was defined as a DE RNA whose p-value from the univariate Cox regression was smaller than 0.05. For each cancer type, the prognostic RNAs were categorized into ceRNAs and non-ceRNAs. The p-values shown in the plot were from the Wilcoxon rank-sum test between hazard ratios of prognostic ceRNAs and non-ceRNAs.

A DE RNA was considered as a potential prognostic biomarker if its Cox proportional hazard ratio’s p-value was smaller than 0.05. Fig 3 shows the hazard ratio distribution of the prognostic ceRNAs versus the prognostic non-ceRNAs for each cancer type. The Wilcoxon rank-sum test was applied to test whether the hazard ratio of prognostic ceRNAs and non-ceRNAs came from the same distribution. In BRCA, we observed a marginal Wilcoxon p-value (0.10). However, the median ceRNAs’ hazard ratio was high (1.54), signifying that an increase of BRCA ceRNAs’ expression was associated with increased risk of death. The Wilcoxon p-values for KIRC (1.4e-35) and HNSC (0.03) were both significant. Notably, in all the three cancer types, compared to the non-ceRNAs’ hazard ratios, the ceRNAs’ hazard ratios were deviated from 1 with higher magnitude, which suggests that the ceRNAs hold higher prognostic power than the non-ceRNAs. We observed that the median hazard ratio of prognostic ceRNAs in KIRC was smaller than 1 whereas the median hazard ratios of prognostic ceRNAs in BRCA and HNSC were higher than 1. This result indicates that the prognostic ceRNAs in KIRC were more likely to be involved in tumor suppressor-related activities, while the prognostic ceRNAs in BRCA and HNSC were more likely to be involved in oncogene-related activities.

#### CeRNA modules were enriched with cancer processes

To examine the biological significance of the inferred ceRNA networks, we clustered each ceRNA network into modules and performed functional enrichment on each module. A ceRNA module was defined as a sub-network of densely connected ceRNAs. We hypothesized that the ceRNA modules, which were extracted from the inferred ceRNA networks, may act as functional units and play an important role in cancer development. To identify ceRNA modules in each ceRNA network, we employed the R package igraph [71] to implement the multilevel graph clustering algorithm [72]. The algorithm identifies densely-connected modules within a network by using a greedy approach that aims to maximize the module’s modularity, which measures the density of connections inside the modules as compared to connections between the modules. In each iteration, each vertex is assigned/reassigned to a module to maximize the module’s modularity. When no vertex can be reassigned, each module is considered as a vertex. The process is restarted and will be stopped when only a single vertex is left or when the modularity can not be increased. Therefore, the algorithm does not require users to specify the number of modules in advance. When applied to large networks (>100k nodes), the algorithm was able to return modules of high modularity without over-merging or over-dividing those modules [72].

To functionally annotate the modules, we performed enrichment analysis between the ceRNAs in each ceRNA module and Cancer Hallmark (CH) terms, Gene Ontology (GO) terms, and KEGG/REACTOME pathways. To make the enrichment test statistically feasible, only modules with at least 10 ceRNAs were used for this analysis. The R package clusterProfiler [73] was used to perform the enrichment analysis.

The number of ceRNA modules containing more than 10 ceRNAs for each cancer type was 18 (BRCA), 11 (KIRC), and 14 (HNSC). The average number of ceRNAs in each module was 74 (BRCA), 87 (KIRC), and 55 (HNSC). Table 6 lists the CH terms that were enriched with the ceRNA modules in each cancer type. Notably, the CH term “Epithelial To Mesenchymal Transition” was enriched in all the three cancer types. The CH terms that were enriched in at least two cancer types included “G2M checkpoint”, “E2F targets”, “TGF beta signaling”, and “MYC Targets V1”. In all the three cancer types, there were several ceRNA modules that were associated with multiple CH terms (i.e., modules 3 and 7 in BRCA, modules 4 and 11 in KIRC, and modules 4 and 7 in HNSC). The same ceRNA modules were also enriched in GO terms and pathways related to regulation of cell division, development, and activation processes (see S2 File). Interestingly, while some ceRNA modules that were not enriched in any CH term, they were enriched in GO terms and pathways associating with disease development and progression processes. For instance, module 15 in BRCA was enriched in the KEGG pathways related to Parkinson, Alzheimer, and Huntington diseases and module 2 in KIRC was enriched in GO Terms involving in negative regulation of metabolic process and molecular function. The list of ceRNAs in each ceRNA module and the list of enriched GO Terms and KEGG/REACTOME pathways for each ceRNA module could be found in S2 File.

**Table 6:** Cancer hallmark terms that were enriched in the ceRNA modules.

Cancer type	Cancer hallmark geneset	Description	Enriched Module
BRCA	Epithelial Mesenchymal Transition	Genes defining epithelial-mesenchymal transition, as in wound healing, fibrosis and metastasis	2, 4, 14
	E2F Targets	Genes encoding cell cycle related targets of E2F transcription factors	3, 7, 13
	Estrogen Response Early	Genes defining late response to estrogen	1, 11
	G2M Checkpoint	Genes involved in the G2/M checkpoint, as in progression through the cell division cycle	3, 7
	TGF Beta Signaling	TGF-beta signaling pathway	6
	Spermatogenesis	Genes up-regulated during production of male gametes (sperm), as in spermatogenesis	7
	IL-6/JAK/STAT3 Signaling	Genes up-regulated by IL6 via STAT3, e.g., during acute phase response	12
	Interferon Gammaresponse	Genes up-regulated in response to IFNG	12
	UV Response Up	Genes up-regulated in response to ultraviolet (UV) radiation	17
KIRC	Epithelial Mesenchymal Transition	Genes defining epithelial-mesenchymal transition, as in wound healing, fibrosis and metastasis	4
	UV Response DN	Genes down-regulated in response to ultraviolet (UV) radiation	4
	Oxidative Phosphorylation	Genes encoding proteins involved in oxidative phosphorylation	11
	MYC Targets V1	A subgroup of genes regulated by MYC—version 1 (v1)	11
	Adipogenesis	Genes up-regulated during adipocyte differentiation (adipogenesis)	11
HNSC	Epithelial Mesenchymal Transition	Genes defining epithelial-mesenchymal transition, as in wound healing, fibrosis and metastasis	4, 5
	TGF Beta Signaling	TGF-beta signaling pathway (UV) radiation	4
	MYC Targets V1	A subgroup of genes regulated by MYC—version 1 (v1)	6
	G2M Checkpoint	Genes involved in the G2/M checkpoint, as in progression through the cell division cycle	7
	E2F Targets	Genes encoding cell cycle related targets of E2F transcription factors	7

### Modification of individual steps in Cancerin pipeline substantially changed the selected ceRNA interactions

In this section, we examine the technical importance of the two major steps in the Cancerin pipeline. The LASSO-based variable selection to select miRNA-mRNA interactions (step 2) and sensitivity correlation-based filtering to select ceRNA interactions (step 3) were two key components in Cancerin. To assess the importance of those two steps, we modified/deactivated those steps to see how it would alter the final ceRNA interaction network topology. Specifically, we kept steps 1 and 3 in Cancerin, but in step 2, we replaced the LASSO-based variable selection procedure by ordinary least square (OLS) multiple regression. For each RNA, its candidate miRNA regulators were selected if their coefficients from OLS were negative and p-values < 0.05. We termed this method “Cancerin (OLS regression)”. We also kept steps 1 and 2 in Cancerin, but in step 3, we deactivated the ceRNA filtering criterion based on sensitivity correlation. We termed this method “Cancerin (sensitivity correlation filtering step deactivated)”. The Cancerin pipeline with no modification is referred to as “Cancerin (original)”.

To compare Cancerin to other existing methods, we replicated the method used in [26, 27], which inferred ceRNA interactions based on negative expression correlation between miRNA and RNA targets and positive expression correlation between RNA targets. We referred to this method as “Correlation-based” method. The method did not consider the other types of regulators besides miRNA (i.e., TF, CNA, and DNA methylation) as potential regulators of gene expression and it also did not take into account the additive effects of multiple regulators on controlling gene expression.

Table 7 summarizes the number of selected ceRNA interactions obtained by applying the “Cancerin (original)”, “Cancerin (OLS regression)”, “Cancerin (sensitivity correlation filtering step deactivated)”, and “Correlation-based method”. As expected, using only expression correlation to infer ceRNA interactions resulted in many ceRNA pairs. Compared to Cancerin, the number of correlation-based ceRNA interactions was more than 6-fold higher in BRCA, 10-fold higher in KIRC, and 6-fold higher in HNSC. All ceRNA interactions found by “Cancerin (original)” were included in the “Correlation-based” method. There were also more ceRNA interactions found by “Cancerin (OLS regression)” than by “Cancerin (original)” but the increased size was smaller compared to the “Correlation-based” method. There is a low overlap between the ceRNA interactions found in “Cancerin original” and the those from “Cancerin (OLS regression)”. Specifically, with respect to interactions found in “Cancerin (original)”, the percentages of common interactions that were also found in “Cancerin (OLS regression)” were 26.8% (BRCA), 40% (KIRC), and 33.2% (HNSC). Compared to “Cancerin (original)”, deactivation of sensitivity correlation filtering step also increased the number of ceRNA interactions. The fold-change increase in each cancer type was 1.7 (BRCA), 4.1 (KIRC), and 3.0 (HNSC). In overall, this comparative analysis indicated that due to several filtering layers used in “Cancerin (original)”, the pipeline is more selective than other methods in selecting ceRNA interactions.

**Table 7:** Number of selected ceRNA interactions by applying different methods.

	BRCA	KIRC	HNSC
Cancerin (original)	4,115	4,639	2,725
Cancerin (OLS regression)	6,039	19,202	6,262
Cancerin (sensitivity correlation filtering step deactivated)	7,018	18,976	8,179
Correlation-based method	25,853	46,518	16,908

We also checked the number of PPIs and TF-gene interactions that were also inferred ceRNA interactions obtained by modifying particular steps in Cancerin or using the “Correlation-based” method. As expected, compared to ceRNA interactions obtained by “Cancerin (original)”, with other methods we observed an increase of ceRNA interactions that were also PPI or TF-gene interactions. Especially the ceRNA interactions inferred by the “Correlation-based” method contained consistently higher percentage of PPI and TF-gene interactions (see S1 Table). These results suggest that the ceRNA interaction predictions obtained from pairwise expression correlation methods could have high false positive rate.

### Inferred ceRNA interactions were able to predict gene expression change

To assess the accuracy of the inferred ceRNA interactions to predict gene expression change, we employed shRNA-mediated perturbation assays data obtained from the Library of Integrated Network-based Cellular Signature (LINCS) database [32]. In the LINCS-L1000 shRNA-perturbation database, gene knockdown experiments using shRNAs were conducted on multiple disease cell lines, making the database a valuable resource to assess gene-gene interactions inferred from computational methods. Each experiment reported gene expression changes of 978 genes as response to the knockdown of a specific gene, which was targeted by a specific shRNA. We referred to the knocked down genes as upstream genes and to the 978 expression-profiled genes as downstream genes. Details of how we used the LINCS-L1000 dataset to evaluate the accuracy of inferred ceRNA interactions in predicting gene expression change were described in S1 Text. In brief, if an upstream ceRNA is silenced, the upstream ceRNA’s miRNA regulators become more available to bind and thereby downregulate the downstream ceRNA partners. Thus, given a downstream ceRNA, its expression level should be lower in response to the silencing of upstream ceRNA partners in comparison to the silencing of other upstream genes. Ratio Fold Change (RFC) of a downstream ceRNA is defined as ratio of its expression fold change following the knockdown of its ceRNA partners to its expression fold change following the knockdown of upstream genes that are not its ceRNA partners. A downstream ceRNA’s RFC was expected to be smaller than 1. Lower value of RFC indicated better prediction of gene expression change due to ceRNA interactions.

Recently, Chiu et al. [74] used the LINCS shRNA-mediated perturbation assays to assess Hermes algorithm, their genome-wide ceRNA interaction prediction tool [29]. We also used the same LINCS dataset (L1000-MCF7) that had been used in [74] to validate our results and to compare accuracy of Cancerin with Hermes. We defined the accuracy of a ceRNA network as the percentage of downstream ceRNAs whose RFCs were smaller than 1. As gene expression in the MCF7 dataset was measured in two different time points (96h and 144h), our analysis was applied on each time point (Table 8). At 96h, out of all downstream ceRNAs (77 in Cancerin and 22 in Hermes), the number of ceRNAs whose RFC was smaller than 1 was 55 in Cancerin (accuracy 71.4%) and 17 in Hermes (accuracy 77.2%). At 144h, out of all downstream ceRNAs (46 in Cancerin and 15 in Hermes), the number of ceRNAs whose RFC was smaller than 1 was 32 in Cancerin (accuracy 69.6%) and 9 in Hermes (accuracy 60%). While overall accuracy (i.e., percentage of total downstream ceRNAs whose RFC was smaller than 1 at both time points) between Cancerin and Hermes was approximately equal (70.7% in Cancerin and 70.2% in Hermes), Cancerin showed consistent accuracy values at both time points. We also computed the RFC values for the downstream ceRNAs obtained when the individual steps in Cancerin pipeline were modified and when only miRNAs were used as potential regulators in the variable selection step (i.e., Cancerin (only_miRNA)). Cancerin outperformed those methods based on the overall accuracy (see Table 8).

**Table 8:** Accuracy of the ceRNA networks inferred by different methods based on LINCS-L1000 (MCF7) dataset.

	Accuracy (96h)	Accuracy (144h)	Overall Accuracy (96h + 144h)
Cancerin (original)	71.4%	69.6%	**70.7%**
Hermes	**77.2%**	60.0%	70.2%
Cancerin (only_miRNA)	67.1%	**73.9%**	69.6%
Cancerin (OLS regression)	66.1%	58.1%	62.9%
Cancerin (sensitivity correlation filtering step deactivated)	66.3%	66.1%	66.2%
Correlation-based method	62.8%	68.2%	65.0%

## Discussion

In this study, we developed Cancerin, a tool to infer genome-wide cancer-associated ceRNA interaction networks and applied it to three types of cancer. Unlike existing ceRNA inference tools that considered miRNAs as the only type of gene regulator, Cancerin considered other types of gene regulators besides miRNAs, namely transcription factors, copy number alteration, and DNA methylation. In addition, using the sensitivity correlation metric proposed in [28], our method directly modeled the ceRNA hypothesis, which posited that the expression profiles of two ceRNAs should be positively correlated and that correlation was conditioned on the expression of their shared miRNA regulators.

The inferred ceRNA networks in all the three cancer types were scale-free networks as the ceRNAs’ degree distribution followed power-law with high fitness. There were very few overlapping interactions between the inferred ceRNA interactions and the PPIs or TF-gene interactions.

Only a subset of input DE RNAs were selected as ceRNAs in the final ceRNA networks. In all three cancer types, the ceRNAs were significantly enriched with cancer-related genes whereas DE RNAs that were not in the ceRNA networks did not have a significant enrichment.

To further explore the biological importance of our inferred ceRNA networks, we clustered ceRNA networks into modules and performed functional enrichment on each module. Various cancer hallmark terms, biological processes, and pathways were enriched in the ceRNA modules across all the three cancer types. In addition, some ceRNA modules were associated with multiple cancer hallmark terms, making the ceRNAs in such modules valuable biomarkers to be further investigated.

To examine the prognostic capability of the inferred ceRNA networks, we performed univariate Cox proportional hazard models for each ceRNA and non-ceRNA. In all the three cancer types, compared to non-ceRNAs, ceRNAs exhibited higher association with cancer outcome. We also observed that KIRC ceRNAs had low hazard ratios indicating that they might act as tumor-suppressors.

We also examined the functional importance of the miRNAs that mediated ceRNA interactions. The miRNAs that mediated the highest number of ceRNA interactions (i.e., let-7a-5p, miR-106b-5p, and miR-9-5p) are well-known in cancer literature; however, their prevalent roles in mediating ceRNA interactions could suggest a novel role in cancer pathogenesis.

Validation of computationally predicted ceRNA interactions is challenging due to the low number of experimentally-validated ceRNA interactions. To address this challenge, we employed the LINCS-MCF7 dataset [32] to check whether the knockdowns of ceRNAs would cause downregulation of their predicted ceRNA partners. We also compared Cancerin’s accuracy with that of Hermes [29], a ceRNA inference tool based on mutual information criterion. Based on the prediction of gene expression change using the inferred ceRNA interactions, Cancerin achieved approximately equal accuracy as Hermes; however the accuracy values from Cancerin at different experimental time points were more consistent.

In summary, we present Cancerin, a computational method that integrates genomic, transcriptomic, and epigenetic regulatory factors to infer genome-wide ceRNA interactions in cancer. Analysis of the inferred ceRNA networks constructed by Cancerin would provide novel insights on the biological functions of this novel layer of gene regulation, especially on how it contributes to cancer pathogenesis.

## Supporting information

S1 File Identified miRNA-RNA interactions and ceRNA interactions in each cancer. (XLSX)

S2 File Functional enrichment analysis results of ceRNA modules. (XLSX)

S1 Table Number of PPIs and TF-gene interactions included in the inferred ceRNA networks. (PDF)

S1 Text Validation of inferred ceRNA interactions using shRNA-perturbation LINCS-L1000 (MCF7) dataset. (PDF)
