# Supplementary material for: Cancerin: A computational pipeline to infer cancer-associated ceRNA interaction networks
Source: PLoS Comput Biol. 2018 Jul 16;14(7):e1006318. doi: 10.1371/journal.pcbi.1006318 (PMC6072113; doi:10.1371/journal.pcbi.1006318)
Supplement: S1 Table — (PDF) [file pcbi.1006318.s003.pdf]

S1 Table: Number of PPIs and TF-gene interactions included in the inferred ceRNA networks

		BRCA	KIRC	HNSC
No. of ceRNA interactions that were also PPIs	Cancerin (original)	35 (0.85%)	29 (0.63%)	20 (0.73%)
	Cancerin (only_miRNA)	42 (1.03%)	45 (0.71%)	42 (1.1%)
	Correlation-based method	257 (0.98%)	358 (0.77%)	177 (1.05%)
	Cancerin (OLS regression)	39 (0.65%)	110 (0.57%)	204 (1.26%)
	Cancerin (sensitivity filtering deactivated)	61 (0.87%)	149 (0.79%)	66 (0.81%)
No. of ceRNA interactions that were also TF-gene interactions	Cancerin (original)	32 (0.78%)	4 (0.09%)	5 (0.18%)
	Cancerin (only_miRNA)	124 (3.03%)	58 (0.92%)	75 (1.96%)
	Correlation-based method	627 (2.43%)	332 (0.71%)	357 (2.11%)
	Cancerin (OLS regression)	39 (0.65%)	33 (0.17%)	60 (0.96%)
	Cancerin (sensitivity filtering deactivated)	41 (0.58%)	31 (0.16%)	59 (0.72%)

The numbers in parentheses indicate the percentage of PPIs and TF-gene interactions in the total number of inferred ceRNA interactions.
