# Supplementary material for: Cancerin: A computational pipeline to infer cancer-associated ceRNA interaction networks
Source: PLoS Comput Biol. 2018 Jul 16;14(7):e1006318. doi: 10.1371/journal.pcbi.1006318 (PMC6072113; doi:10.1371/journal.pcbi.1006318)
Supplement: S1 Text — (PDF) [file pcbi.1006318.s004.pdf]

S1 Text: Validation of inferred ceRNA interactions using shRNA-perturbation LINCS-L1000 (MCF7) datatset

1 Description of shRNA-perturbation LINCS-L1000 (MCF7) dataset

In the LINCS-L1000 shRNA-perturbation database, each gene knockdown experiment involved using a specific shRNA to target and thereby silenced a gene [1]. The shRNAs were designed to target and silence its predetermined target (i.e., to avoid off-target matching and unwanted miRNA effects). For each experiment, expression of 978 landmark genes were profiled before and after the gene knockdown. Thus, in response to a gene knockdown experiment, for each of the 978 genes, its expression fold change (EFC) and p-value from differential expression analysis were reported. As mentioned in the article, we refer to the targeted/knocked-down genes as upstream genes and the 978 genes as downstream genes.

We employed data from LINCS-L1000 shRNA-perturbation performed on the breast cancer cell MCF7. In the MCF7 data set, expression changes of the 978 downstream genes were recorded at two different time points (96h and 144h). Thus, our analysis was specific for each time point. One upstream gene could be silenced by multiple shRNAs (on average by 3 shRNAs). Consequently, a downstream gene would have multiple EFC records corresponding to the silencing of the upstream gene. In such cases, we used the downstream gene's EFC average to represent its overall EFC. The number of upstream-downstream pairs in each time point were 2,578,986 pairs (96h) and 1,022,988 pairs (144h). The number of upstream genes in each time point were 2,637 (96h) and 1,046 (144h)

2 Assessing accuracy of using inferred ceRNA interactions to predict gene expression change in LINCS-L1000 (MCF7) data

We used LINCS-L1000 (MCF7) shRNA-perturbation data to assess if the inferred ceRNA interactions can be used to predict gene expression patterns. We expected that if a downstream gene is an inferred ceRNA, its EFC would be lower in response to the silencing of its upstream ceRNA partners, compared to the silencing of its upstream non-ceRNAs. In other words, for a downstream ceRNA gene, its ratio of expression fold change is expected to be smaller than 1 (see Eq.1).

Given the inferred ceRNA interaction results, a downstream $ceRNA_i$ in MCF7 has M upstream ceRNA partners and N upstream non-ceRNAs. Let $EFC(ceRNA_i \leftarrow ceRNA_m)$ and $EFC(ceRNA_i \leftarrow RNA_n)$ be the expression fold change of $ceRNA_i$ caused by silencing of its ceRNA partner $ceRNA_m$ and the

non-ceRNA RNA_n, respectively. The ratio of expression fold change $RFC(ceRNA_i)$ was defined in Eq. 1.

$$RFC(ceRNA_i) = \frac{\frac{1}{M}\sum_{m=1}^{M} EFC(ceRNA_i \leftarrow ceRNA_m)}{\frac{1}{N}\sum_{n=1}^{N} EFC(ceRNA_i \leftarrow RNA_n)} \quad (1)$$

Lower RFC indicates better prediction of gene expression change due to inferred ceRNA interactions.

In the LINCS-L1000 (MCF7) dataset, a subset of all upstream genes and a subset of all downstream genes were also inferred ceRNAs. A downstream ceRNA was selected for this analysis if it had at least one upstream ceRNA in the MCF7 dataset. As the DyCeR algorithm only selected ceRNA interaction out of all possible pairs between DE mRNAs, we only kept the upstream genes that were also DE mRNAs in the TCGA-BRCA dataset.

References

1. Liu C, Su J, Yang F, Wei K, Ma J, Zhou X. Compound signature detection on LINCS L1000 big data. Molecular BioSystems. 2015;11(3):714–722.
